# Genome Sequence of *Janthinobacterium* sp. CG23_2, a Violacein-Producing Isolate from an Antarctic Supraglacial Stream

**DOI:** 10.1128/genomeA.01468-15

**Published:** 2016-01-28

**Authors:** Heidi J. Smith, Christine M. Foreman, Tatsuya Akiyama, Michael J. Franklin, Nicolas P. Devitt, Thiruvarangan Ramaraj

**Affiliations:** a Center for Biofilm Engineering, Montana State University, Bozeman, Montana, USA; b National Center for Genome Resources (NCGR), Santa Fe, New Mexico, USA

## Abstract

Here, we present the draft genome sequence for the violacein-producing *Janthinobacterium* sp. CG23_2 isolated from an Antarctic supraglacial stream. The genome is ~7.85 Mb, with a G+C content of 63.5%. The genome includes 7,247 candidate protein coding genes, which may provide insight into UV tolerance mechanisms.

## GENOME ANNOUNCEMENT

Organisms inhabiting Antarctic supraglacial environments are subjected to a variety of environmental stresses. Ozone depletion over Antarctica has altered the spectral composition of solar radiation (1), and harmful UVC (100 to 280 nm) radiation is present on ice surfaces (2). Inescapable to microorganisms throughout the course of the austral summer, UV radiation damages biological macromolecules, including nucleic acids, lipids, and proteins and may lead to cell death. In order for microorganisms to inhabit environments with high UV radiation, efficient protective mechanisms and DNA and protein repair mechanisms are necessary. One mechanism for the protection from UV induced biological damage is for cells to produce protective pigments (3, 4).

The purple pigment violacein is produced by certain members of the β-proteobacteria, including some *Janthinobacterium* strains. Violacein has been investigated for its antimicrobial (5), antioxidant (6), and UV protection properties (4). Although the exact mechanism of violacein protection against UV damage is currently not well understood, evidence suggests that violacein can detoxify free radicals induced by UV radiation (4).

*Janthinobacterium* spp. are found in many different environments, including lakes, soils, and glaciers (7–9). We recently reported the genome sequence of a nonpigmented *Janthinobacterium* sp. isolated from a supraglacial stream. Here we present the genome sequence of a violacein producing strain *Janthinobacterium* sp. CG23_2, isolated from the same system in Antarctica. Genomic analyses of these strains will offer insight into UV tolerance mechanisms from environmentally relevant isolates.

*Janthinobacterium* sp. strain CG23_2 was isolated from the Cotton Glacier in the Antarctic Dry Valleys (77° 07S, 161° 50E). The organism was isolated on R2A agar medium incubated in the dark at 4°C for 12 days. *Janthinobacterium* sp. strain CG23_2 is a psychrotolerant, aerobic, violacein-pigmented, rod-shaped, Gram-negative, catalase-positive organism. Genomic DNA was isolated following standard cetryltrim-ethylammonium bromide (CTAB) isolation protocols (http: //www.jgi.doe.gov).

Whole-genome DNA sequencing was performed using a Pacific Biosciences (PacBio, Menlo Park, CA) RS II instrument (10). A single molecule real-time (SMRT) cell library was constructed with 10 *μg* input DNA using the PacBio 20-kbps protocol. The library was then loaded onto one SMRT cell and sequenced using P5 polymerase and C3 chemistry with 180-min movie times. Sequencing yielded a total of 99,287 reads with mean read length of 5.9 kbps, totaling 581,513,481 bps (≈85-fold coverage). *De novo* assembly was constructed using the hierarchical genome assembly process (HGAP2) protocol from SMRT Analysis v2.0, including consensus polishing with Quiver (11, 12). The final assembly consists of four contigs with a total genome size of ≈7.85 Mbps. Approximately 93% of the genome is contained within two large contigs (4.2 and 3.1 Mbps). Remaining sequences were divided into two smaller contigs ranging from 420 to 153 kbps. A total of 7,247 candidate protein-coding genes were predicted using RAST (13) with a total G+C content of 63.5%. Upon comparison with genomes available within the RAST the closest relative to *Janthinobacterium* sp. CG23_2 was determined to be *Janthinobacterium* sp. Marseille (score 542).

### Nucleotide sequence accession numbers.

This genome sequence has been deposited in EMBL/GenBank under the accession number CYSS00000000. The version described in this paper is the first version, CYSS00000000.1.
